# Vascular endothelial growth factor levels and rheumatic diseases of the elderly

**DOI:** 10.1186/s13075-016-1184-x

**Published:** 2016-12-01

**Authors:** Perrine Smets, Valérie Devauchelle-Pensec, Paul-Olivier Rouzaire, Bruno Pereira, Marc Andre, Martin Soubrier

**Affiliations:** 1 Département de médecine interne, Centre Hospitalier Universitaire Gabriel Montpied, 58, rue Montalembert, 63000 Clermont-Ferrand, France; 2 Département de rhumatologie, Centre Hospitalier Régional et Universitaire de Brest, 2 Avenue Foch, 29200 Brest, France; 3 Département d’immunologie, Centre Hospitalier Universitaire Gabriel Montpied, 58, rue Montalembert, 63000 Clermont-Ferrand, France; 4 Département de délégation de recherche clinique, Centre Hospitalier Universitaire Gabriel Montpied, 58, rue Montalembert, 63000 Clermont-Ferrand, France; 5 Département de rhumatologie, Centre Hospitalier Universitaire Gabriel Montpied, 58, rue Montalembert, 63000 Clermont-Ferrand, France

**Keywords:** Vascular endothelial growth factor, Rheumatoid arthritis, Polymyalgia rheumatica, Giant cell arteritis, RS3PE syndrome

## Abstract

**Background:**

Increasing vascular endothelial growth factor (VEGF) has been reported in remitting symmetrical seronegative synovitis with pitting edema (RS3PE) syndrome, rheumatoid arthritis (RA), polymyalgia rheumatica (PMR) and giant cell arteritis (GCA). The aim of this study was to compare VEGF levels in patients over 60 years of age who have RS3PE, RA, PMR or GCA so as to determine whether elevated VEGF is specific for a rheumatic disease, the inflammation or edema that occurs with these pathological conditions.

**Methods:**

In this retrospective, multicentric study we assessed serum and plasma levels of VEGF in patients over 60 years of age with rheumatic diseases that were either *de novo* or of recent onset according to the initial clinical presentation, and we compared these patients with a control group.

**Results:**

Serum and plasma VEGF levels were determined in 80 patients (5 with RS3PE, 13 with RA, 44 with PMR, and 18 with GCA) and 37 controls. Edema occurred in five patients with RS3PE, four with RA, and one with PMR, but not patients with GCA. Serum VEGF levels were significantly higher in individuals with rheumatic diseases (849 (405.5–1235.5) pg/ml) relative to the controls (484 (302–555) pg/ml) (*p* < 0.001). There were no significant differences between patients with RS3PE, RA, PMR, or GCA in terms of the VEGF serum levels (*p* = 0.60) or plasma levels (*p* = 0.57). Similarly, the occurrence of edema did not correlate with VEGF levels.

**Conclusion:**

VEGF increases in rheumatic diseases compared to a control group. This was not associated with specific rheumatic diseases or with edematous rheumatic diseases.

## Background

Rheumatic diseases in individuals over the age of 60 years are difficult to diagnose in light of their non-specific clinical presentation. Polymyalgia rheumatica (PMR), giant cell arteritis (GCA), and remitting symmetrical seronegative synovitis with pitting edema (RS3PE) are pathological conditions that are specific to elderly individuals, although rheumatoid arthritis (RA) is the most common of the rheumatic diseases in this population [1]. While polymyalgia symptoms are seen in PMR [2] and RS3PE [3], these also occur in 40–60% of patients with GCA [2] and in an isolated manner in 25% of patients newly diagnosed with RA [1]. Similarly, while pitting edema of both hands is ubiquitous in RS3PE [3], this may be encountered in 12% of patients with PMR [4], 10% of patients with RA [1], and sometimes in patients with GCA [2]. Pease et al. performed longitudinal follow up of 349 patients diagnosed with GCA (*n* = 57, (16.5%)), PMR (*n* = 147, (42%)), or RA (*n* = 145 (41.5%)), over a period of at least two years. At the end of this follow-up period, 10% of the patients were reclassified [5].

Vascular endothelial growth factor (VEGF) is a growth factor for the vascular endothelium that has key roles in angiogenesis and vascular permeability. It is synthesized by various endothelial cells, fibroblasts, smooth muscle cells, and macrophages, and it accumulates in platelets [6]. Elevated VEGF has been reported in RS3PE relative to patients with other connective tissue disorders (such as RA, systemic lupus erythematosus, mixed connective tissue disease, polymyositis, and dermatomyositis) and healthy individuals, and this can result in edema [7].

The aim of this study was to assess VEGF in patients over 60 years of age, who had RS3PE, RA that was starting to exhibit symptoms of polymyalgia and/or edema, PMR, or GCA, so as to determine whether VEGF is specific to inflammatory pathological change in aged individuals or to the edema seen with these conditions.

## Methods

We carried out a retrospective, multicentric study in two rheumatology departments and one internal medicine department. We included patients who were 60 years of age or older, and who had new or recent onset (<12 months) rheumatic disease; with RA corresponding with the criteria for RA according to the American College of Rheumatology (ACR) 1987 and ACR/European League Against Rheumatism (EULAR) 2010, PMR according to the criteria of Chuang and Hunder, GCA according to the ACR criteria, or RS3PE according to the McCarthy description [3]; and in whom serum and/or plasma VEGF had been determined between the 1 January 2002 and the 31 December 2015. We excluded patients receiving steroidal anti-inflammatory and/or immunosuppressive drugs and/or biotherapy before the determination of VEGF.

We identified a control group of patients >60 years of age, who had VEGF determined in the same time period and who did not have rheumatic disease other than microcrystalline arthritis. In both groups, we excluded patients with pathological conditions or health conditions known to involve VEGF, such as the polyneuropathy, organomegaly, endocrinopathy, monoclonal plasma-proliferative disorder, and skin changes (POEMS) syndrome [8], solid tumors, and hematological malignancies [9, 10]. The characteristics and the laboratory results for the patients were recorded in their medical files. Serum and plasma VEGF were determined in the immunology laboratory of University Hospital Center of Clermont-Ferrand, FRANCE, using an immuno-enzymatic method and a kit from RnDSystems: Quantikine® ELISA. This allowed for quantitative determination of the VEGF 165 isoform of VEGF A. The normal range for serum VEGF is 62–707 pg/ml, and for plasma VEGF it is lower than 115 pg/ml.

Investigation of the data was approved by the *Comité de Protection des Personnes Sud-Est* 6 (number 2015/CE 61), number IRB 00008526 and the *Comité de Protection des Personnes Ouest* 6 – 704, the CIC 1412 and the CRB Santé de Brest BB-0033-00037.

Statistical analysis was performed using Stata software, version 13 (StataCorp, College Station, TX, USA). The tests were two-sided, with a type I error set at α = 0.05. Quantitative data were presented as mean ± standard deviation or median (interquartile range) according to statistical distribution (assumption of normality studied by the Shapiro-Wilk test). Comparisons between the independent groups (i.e. patients with RA, RS3PE, PMR, or GCA) were performed by analysis of variance (anova) or the Kruskal-Wallis (KW) test if the assumptions of anova were not met (normality, homoscedasticity studied using the Bartlett test). When appropriate (omnibus *p* value <0.05), a suitable post-hoc test was used: Tukey-Kramer post anova and Dunn post KW. Comparisons of categorical variables between independent groups were performed using the chi-squared or Fisher’s exact test, followed by the Marascuilo procedure for multiple comparisons. The study of relationships between quantitative parameters (VEGF, erythrocyte sedimentation speed, C-reactive protein (CRP), platelets count) was performed using Spearman correlation coefficient, according to statistical distribution of the studied parameters. Last, random-effect multivariate regression analysis was used to study the relationship between VEGF and different pathological conditions taking into account adjustment for the gender and age of the patients, the erythrocyte sedimentation rate (ESR), and the platelet counts. In these models, the effect of center was considered as a random-effect.

## Results

Eighty patients with rheumatic diseases were assessed (Table 1). This sample comprised 44 women and 36 men (sex ratio of 1.2) with a mean of 73 years of age. There were 5 patients diagnosed with RS3PE, 13 with RA, 44 with PMR, and 18 with GCA. A median delay of 4 months was required for the classification of the rheumatic disease in nine patients. The median follow-up duration was 32.67 months (±25.04). There were 65 patients (81.2%) with polymyalgia symptoms, and 10 patients (12.5%) had an edematous form. The five patients with RS3PE syndrome were afflicted by symptoms of polymyalgia and edema. Among the 13 patients with RA, 5 (38.5%) were seropositive, 2 had erosive disease, 8 (61.5%) had symptoms of polymyalgia, and 4 (30.7%) had edema. The 44 patients with PMR had polymyalgia symptoms and only 1 patient had an edematous form. Out of the 18 patients with GCA, 14 (77.7%) had a positive temporal artery biopsy, 8 (44.4%) had associated polymyalgia symptoms, and none had an edematous form.

**Table 1:** Characteristics of patients with a rheumatic disease and the controls

	Rheumatic disease (*n* = 80)	Control (*n* = 37)	*P* value (patients vs controls)
Age, years, mean ± SD	73.05 ± 8.03	73.35 ± 8.55	0.98
Sex, female/male	44/36	24/13	0.31
ESR, mm/h, mean ± SD	65.11 ± 32.01	35.60 ± 25.89	**<0.001**
CRP, mg/L, mean ± SD	80.79 ± 74.05	12.83 ± 19.92	**<0.001**
Platelets, Giga/L, mean ± SD	371.89 ± 119.56	262.50 ± 89.59	**<0.001**
Serum VEGF, pg/ml, median (ITQ)	849 (450.50–1235.50)	484 (308–555)	**<0.001**
Plasma VEGF, pg/ml, median (ITQ)	86 (50.50–148.50)	67 (43–105)	0.16

*SD* standard deviation, *ESR* erythrocyte sedimentation rate, *CRP* C-reactive protein, *VEGF* vascular endothelial growth factor, *ITQ* interquartile

Bold is significant value

The control group comprised 37 patients, of whom 25 were women and 13 men (sex ratio of 1.9) with a mean age of 73 years. They were being monitored for a monoclonal gammopathy of undetermined significance (*n* = 11), arthrosis (*n* = 6), a narrowing of the lumber vertical canal (*n* = 3), fibromyalgia (*n* = 3), lumbosciatica (*n* = 2), microcristalline arthritis (*n* = 4), peripheral neuropathy (*n* = 5), osteoporotic fracture (*n* = 2), and for papillitis (*n* = 1).

CRP, ESR, the platelet count, and serum VEGF were significantly higher (*p* < 0.001) in individuals with rheumatic disease than in the controls (Table 1). On the other hand, there was no difference in plasma VEGF (*p* = 0.24).

There were no significant differences in serum VEGF between patients with RS3PE, RA, PMR, or GCA (*p* = 0.60) or in plasma VEGF (*p* = 0.57) (Table 2). By multivariate analysis of the patients with rheumatic diseases adjusting for sex, ESR, and platelet count, serum and plasma VEGF did not differ according to the rheumatoid diagnosis. There was no difference in serum VEGF or plasma VEGF in patients with rheumatic disease with or without edema.

**Table 2:** Characteristics and levels of VEGF in the various rheumatic diseases

	RS3PE (*n* = 5)	RA (*n* = 13)	PMR (*n* = 44)	GCA (*n* = 18)	*P* value	Edematous forms (*n* = 10)	Non-edematous forms (*n* = 70)	*P* value
Age, years, mean ± SD	76.4 ± 9.56	71 ± 7.97	71.41 ± 7.43	76.44 ± 8.07	0.17	76 ± 8.42	72.63 ± 7.95	0.21
Sex, female/male	2/3	8/5	19/25	15/3	0.02^a^	5/5	39/31	0.74
ESR, mm/h, mean ± SD	50.40 ± 29.84	61.67 ± 39.55	61.24 ± 30.35	82.44 ± 27.34	0.03^b^	54.89 ± 31.14	66.50 ± 32.10	0.31
CRP, mg/L, mean ± SD	57 ± 35.95	113.62 ± 121.12	73.07 ± 63.11	82.63 ± 62.50	0.81	76.33 ± 54.91	81.38 ± 76.53	0.84
Platelets, Giga/L, mean ± SD	309.60 ± 58.796	334.33 ± 114.20	313.58 ± 104.71	432.56 ± 96.32	0.03^c^	314.33 ± 63.69	379.29 ± 123.29	0.12
Serum VEGF, pg/ml, median (ITQ)	858 (801–1048)	590 (303–1117)	859.5 (413-1386.5)	882 (671–1131)	0.60	953 (712–1927)	844 (436–1160)	0.34
Plasma VEGF, pg/ml, median (ITQ)	79 (52–94)	90 (47–148)	67 (48–160.5)	120 (69–181)	0.57	108.5 (80.75–146.75)	78 (47–149)	0.58

^a^Significant difference (*p* = 0.02) between PMR and RA on the one hand and PMR and GCA on the other hand; ^b^significant difference (*p* = 0.03) between GCA and RS3PE on the one hand and GCA and PMR on the other hand; ^c^significant difference (*p* = 0.03) between GCA and RA on the one hand and GCA and RS3PE on the other hand. *VEGF* vascular endothelial growth factor, *RS3PE* remitting symmetrical seronegative synovitis with pitting edema, *RA* rheumatoid arthritis, *PMR* polymyalgia rheumatica, *GCA* giant cell arteritis, *SD* standard deviation, *ITQ* interquartile, *ESR* erythrocyte sedimentation rate, *CRP* C-reactive protein

**Fig. 1:**
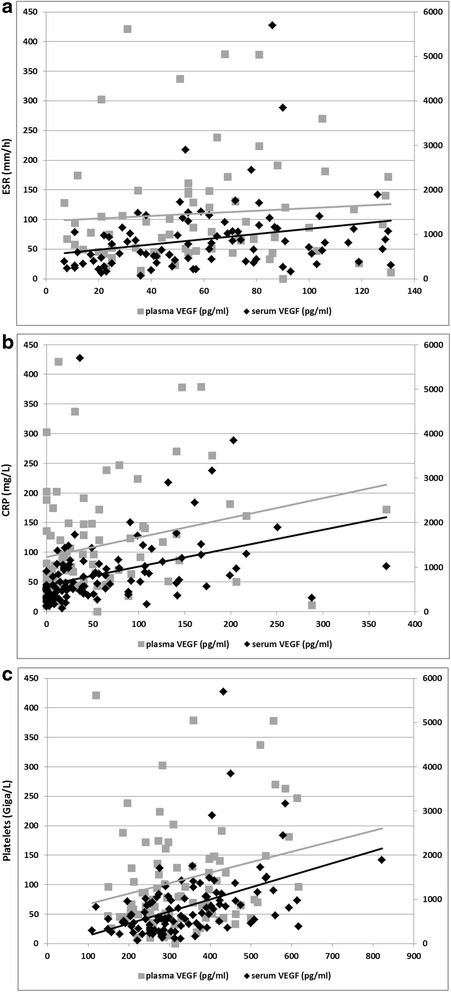
Correlation between serum and plasma vascular endothelial growth factor (*VEGF*) and the markers of inflammation, erythrocyte sedimentation rate (*ESR*) (**a**), C-reactive protein (*CRP*) (**b**), and platelets (**c**) in the 117 patients in the study. **a** Plasma VEGF and ESR (*r* = 0.12, *p* = 0.34), and serum VEGF and ESR (*r* = 0.31, *p* = 0.003). **b** Plasma VEGF and CRP (*r* = 0.30, *p* = 0.005), and serum VEGF and CRP (*r* = 0.51, *p* < 0.001). **c** Plasma VEGF and platelets (*r* = 0.20, *p* = 0.06), and serum VEGF and platelets (*r* = 0.50, *p* < 0.001)

There was positive correlation between serum VEGF and inflammation markers on the one hand, the ESR (*r* = 0.28, *p* = 0.03) and CRP (*r* = 0.38, *p* < 0.002), and the platelet count (*r* = 0.47, *p* < 0.001) on the other hand. Plasma VEGF, by contrast, was correlated with CRP (*r* = 0.30, *p* = 0.005) (Fig. 1).

## Discussion

Our study has shown that serum VEGF increases in rheumatic disease in elderly individuals irrespective of the specific diagnosis (i.e. RS3PE, RA, PMR, or GCA) relative to control subjects. This increase in serum VEGF could specifically indicate an inflammatory state as it correlated with increases in ESR, CRP, and the platelet count, which are all markers of inflammation [11]. We did not find an association between VEGF and edematous rheumatic disease.

Making a clinical diagnosis of rheumatic disease in elderly individuals can prove difficult, particularly with isolated symptoms of polymyalgia [5]. In a study of three patients with RS3PE, Arima et al. reported an association with serum VEGF levels, with a mean increase of 2,223.3 pg/ml, relative to other connective tissue disorders (such as RA, systemic lupus erythematosus, mixed connective tissue disease, polymyositis, dermatomyositis), suggesting that RS3PE can be classified as a VEGF-associated disorder, which can lead to edema [7].

Another study, assessing serum VEGF in RA identified higher levels in 22 individuals with RA, with a mean age of 39 years, relative to 10 healthy subjects (*p* < 0.01) and or 10 patients with arthrosis (*p* < 0.05) [12]. VEGF was also reported in 29 patients with PMR, relative to 20 age and gender matched controls. Median serum VEGF was 458 (53–1362) pg/ml in the patients and 172 (20–514) pg/ml in the controls (*p* < 0.001) [13]. In GCA, Baldini et al. identified an increase in serum VEGF in 75 patients relative to 24 controls (683.18 ± 69.74 pg/ml vs 226.35 ± 53.66 pg/ml, *p* < 0.001) and relative to 15 patients with RA (294.05 ± 56.49 pg/ml, *p* < 0.039) [14]. Our study is the only one to compare the involvement of serum and plasma VEGF in these rheumatic diseases (i.e. RS3PE, RA, PMR, and GCA). Unfortunately, VEGF is not a marker of specific rheumatic disease, as has been suggested previously. It is also not associated with edematous rheumatic disease [7].

Discordance between plasma and serum VEGF have been previously reported [15]. In vitro it has been attributed to the release of VEGF stored in platelets during the processing of serum. Thus, as plasma is generally collected in a tube with anticoagulant, the platelets are less active and contribute less to the level of VEGF that is measured [8]. We have found a correlation between serum VEGF and the platelet count, and such correlation was not seen with plasma VEGF. This is in keeping with other reports [15]. There remains uncertainty as to which VEGF measurement is the most relevant. With POEMS syndromes, where VEGF is a diagnostic criterion, Tokashiki et al. recommend using serum rather than plasma VEGF, thus, allowing analysis of all of the blood constituents [15]. However, a degree of variability has been reported in serum VEGF that is not seen with plasma VEGF. This could depend on the processing of the blood, with more pronounced release of VEGF by platelets depending on the coagulation time of the sample, and on the analysis technique [8, 15].

One of the limitations of our study is, first of all, its retrospective nature. Determination of serum and plasma VEGF is performed routinely in our medical center in older individuals with inflammatory rheumatic disease that is hard to characterize. The studied population is hence not representative of the rheumatic diseases in this age group. In fact our patients with rheumatic disease mainly have polymyalgia symptoms (81.2%). Our subgroup of patients with RA does not include the typical seropositive or erosive RA, which are easier to diagnose and which represent 70% of RA in elderly individuals [1]. Therefore, the VEGF results obtained in our patients with RA do not reflect the actual VEGF levels in patients with RA who are over 60 years of age.

## Conclusions

Our study demonstrates that serum VEGF increases in elderly patients with inflammatory rheumatic disease compared to a control group. This is neither associated with specific rheumatic diseases nor with edematous rheumatic disease. Our results only indicate that VEGF is a parameter associated with inflammation.

## Abbreviations

CRP: C-reactive protein
ESR: erythrocyte sedimentation rate
GCA: giant cell arteritis
ITQ: interquartile
KW: Kruskal-Wallis
PMR: polymyalgia rheumatica
RA: rheumatoid arthritis
RS3PE: remitting symmetrical seronegative synovitis with pitting edema
SD: standard deviation
VEGF: vascular endothelial growth factor
